# Evo-Inspired Engineering of Radical Phenotypes and Emergent Traits

**DOI:** 10.1093/icb/icaf107

**Published:** 2025-08-06

**Authors:** Leslie S Babonis, Daniel A Hahn, Allen P Liu, Joshua R Widhalm

**Affiliations:** Department of Ecology and Evolutionary Biology, Cornell University, Ithaca, NY 14853, USA; Department of Entomology and Nematology, University of Florida, Gainesville, FL 32611, USA; Department of Mechanical Engineering, University of Michigan, Ann Arbor, MI 48109, USA; Center for Plant Biology and Department of Horticulture and Landscape Architecture, Purdue University, West Lafayette, IN 47907, USA

## Abstract

Nature has already solved many challenges that synthetic biology seeks to address. At the same time, high-throughput platforms and artificial intelligence-driven design tools are redefining the landscape of synthetic biology. By integrating evolutionary insights with new enabling technologies, we are poised to move beyond modifying existing traits to engineering entirely new cellular functions. This series of vignettes explores how phenotypic engineering, inspired by nature’s most extreme adaptations, represents the next frontier in synthetic biology. We first make a case for the importance of continuing to explore nature by examining how foundational discoveries in molecular biology, motivated by curiosity to understand how life works rather than immediate application, laid the groundwork for modern applications in biology. Next, we discuss how evolution produced novel cell types with extraordinary properties and how leveraging these innovations can enable the creation of radical phenotypes beyond natural evolutionary constraints. Then, we explore the potential of artificial cells, constructed from the bottom up, as experimental platforms for studying genotype–phenotype relationships and testing evolutionary principles in real time. Finally, we argue that engineering radical phenotypes and emergent traits will require better investment in basic science, infrastructure, and graduate training to avoid bottlenecking innovation. History has shown that committing to basic science results in transformative solutions with far-reaching societal and economic benefits. By drawing inspiration from evolution and expanding synthetic biology beyond traditional model systems, we can push current boundaries to open new avenues for fundamental discovery and technological advances to stimulate the bioeconomy.

## Introduction

(*by Daniel A. Hahn*)

We are rarely smart enough to set about on purpose making the discoveries that will drive our economy and safeguard our lives. Often, we lack the fundamental research. Instead, we pursue a broad range of investigations of Nature, and applications we never dreamed of emerge. Not always, of course. But often enough.

-Carl Sagan, The Demon-Haunted World: Science as a Candle in the Dark

Fundamental research in the biological sciences has frequently been translated into practical products that improve human lives. From the discovery of penicillin that revolutionized modern medicine (Gaynes 2017) to fundamental studies of the *Bacillus thuringiensis* (Bt) toxin that led to the development of insect-resistant Bt crops (Gassmann and Reisig 2023), basic research discoveries that were not initially expected to yield applications have paid off big for society (Ding et al. 2006; Gaynes 2017). These discoveries not only enhanced human health and well-being, but generated billions of dollars in economic benefits (Wong 1996; Moyo and Phiri 2024). Enabling creative science to be driven by the creativity and commitments of its practitioners, not the prescriptions of a regulatory body, is an important way to ensure the future of such discoveries. Here, we aim to inspire greater investment in basic research that leverages our knowledge of natural history to purposefully engineer novel phenotypes, an important facet of the field of synthetic biology. Studying the emergent traits produced when cellular and organismal phenotypes that do not occur together in nature are combined using the tools of synthetic biology will reveal fundamental insights into natural processes and provide new avenues to test what limits the evolution of novelty. Even our most creative minds have not been able to fully predict the solutions that nature has come up with for solving various biotic and abiotic challenges. Thus, remaining open-minded to what Nature may teach us will provide fodder for many potential future applications to bolster the bioeconomy and improve the human condition.

Humans have intentionally and unintentionally shaped the phenotypes of other organisms for millennia. Classical selective breeding has brought us nearly all of our food plants and animals, as well as many varieties of plants and animals whose phenotypes have been tuned for their aesthetic value, from award-winning roses to Yorkshire terriers. Efforts at classical breeding have increasingly used genomic markers to improve the efficiency and specificity of selection in classical quantitative genetic designs (Meuwissen et al. 2001; Varshney et al. 2021). In addition to classical breeding, genetic engineering approaches have fundamentally increased our ability to manipulate organismal phenotypes in both agriculture and biomanufacturing. For example, the production of human insulin in bacterial expression systems in the 1980s massively expanded the availability and affordability of this critical drug (Quianzon and Cheikh 2012). Similarly, transgenic insertion of a bacterial protein that kills insect pests into major crop plants in the 1990s, most notably corn and soybeans, has transformed yields for these key crops while enhancing some ecosystem services lost when traditional chemical pesticides are used (Dively et al. 2018; Lu et al. 2022). For example, controlling insect pests of corn with area-wide adoption of Bt maize, also reduces the incidence of pests in nearby vegetable plots, including organic plots, reducing the frequency of insecticide applications needed for pest management in these vegetable fields within the region (Dively et al. 2018). Despite these successes and massive investment into genetic engineering approaches in agriculture, biomedicine, and biomanufacturing, transgenic engineering of organismal phenotypes has only recently been assimilated into fundamental studies of biology, such as ecology and evolutionary biology. Application of genetic engineering approaches to comparative biology will expand our understanding of fundamental questions in cell biology and physiology. Here, we emphasize that investment is needed to democratize genetic engineering and synthetic biology to study novel phenotypes outside of the currently narrow communities in which these tools and perspectives are currently employed.

Manipulations that we consider forms of phenotypic engineering have been used to test hypotheses in ecology and evolution for many decades. For example, David Lack’s classical studies of adding or subtracting eggs to manipulate the clutch sizes of birds to test life-history tradeoffs between offspring size and number in the 1940s were an early and simple form of phenotypic engineering that had an enormous impact on the way that evolutionary ecologists approached testing hypotheses (Lack 1947, 1948, 1954). Similarly, phenotypic manipulation of morphological traits has long been used to test evolutionary ecology hypotheses, from altering the length of male widowbird tails by pasting on additional feathers to test principles of sexual selection (Andersson 1982) to removing leaves from plants to test for effects of leaf area and photosynthetic capacity on seed production (Willson and Price 1980). Surgical and endocrine manipulations have also been used to engineer phenotypes to test hypotheses in both laboratory and field settings. The most powerful of these approaches combine methods to tune phenotypes quantitively, both increasing and decreasing traits around population means, as well as pushing individual traits beyond current population ranges. For example, Sinervo and colleagues famously combined surgical ablation of egg follicles and endocrine manipulation of egg production, as well as physically altering quantities of yolk in eggs, in a series of studies that revolutionized the testing of hypotheses about clutch size and offspring numbers in lizards (Sinervo and McEdward 1988; Sinervo and Licht 1991; Sinervo 1996). There are many such powerful examples of organismal-level phenotypic engineering approaches being used for testing evolutionary ecology hypotheses in non-model organisms, and sadly, it is beyond our scope to cover all of the great examples here. However, our point is that highly controlled phenotypic engineering studies can lead to enormous advancements in our understanding of fundamental processes.

Integrative biology is currently undergoing a rapid expansion of tools and techniques that are revolutionizing phenotypic engineering approaches via genetic manipulations. Genetic approaches have been successfully applied in a cadre of model organisms to engineer phenotypes for decades. These approaches range from simple mutagenesis screens that produced loss-of-function mutants to carefully regulated conditional overexpression of candidate genes and even swapping out different alleles at a locus to test for allele-specific effects of candidate loci. These genetic approaches are most often deployed in model organisms for specific tests of gene function, but have also been used to test ecological and evolutionary hypotheses in model organisms as well (Gilmour et al. 2000; McCullough et al. 2022). Loss-of-function mutants are becoming increasingly accessible to researchers in many facets of biology using a variety of organisms (Crawford et al. 2020; Short et al. 2020). Techniques that overexpress specific candidate genes have been slower to transition into engineering phenotypes in non-model or emerging-model organisms, although some good examples do exist (Teets et al. 2019; Foster et al. 2024). Similarly, swapping specific alleles within a genetic background to test for allelic effects on phenotypes has become standard in model organisms like *Drosophila melanogaster* (Lamb et al. 2017; Hoedjes et al. 2023), and this approach is poised to trickle out into non-model organisms, beginning often with *in vitro* studies of protein function or cell culture experiments as a start to understanding functions in whole organisms (Cziczo et al. 2013). Some difficulties in expanding the above techniques into more organisms are a lack of tools and infrastructure for training in genetic engineering, as well as the difficulties of laboratory culturing and the long duration of some organismal lifecycles making transgenics prohibitively time consuming. All of these examples make use of genetic engineering technology to modify existing phenotypes. While valuable, to reach the real promise of genetic manipulation for phenotypic engineering we require the ability to easily introduce novel candidate genes into the genomes of a broad swath of non-model organisms. This ability will enable testing of hypotheses about gene function in a variety of genetic backgrounds and allow the study of the adaptive value of the novel phenotypes produced.

Making radical changes in phenotypes will require the ability to perform transgenic conditional overexpression of multiple genes or even entire gene networks both within and across species. For example, many plants require insect pollinators to reproduce effectively. In wild plant populations, pollen limitation due to the unavailability of pollinators can limit reproduction and thus, the persistence of plant populations (Burns et al. 2019; Siopa et al. 2023). Perhaps even more obviously, many cultivated crop plants require growers to bring in supplemental pollinators such as honeybees or solitary bees to maximize crop yields (Gallai et al. 2009; Khalifa et al. 2021). One such pollinator that growers can use to pollinate their crops is the alfalfa leafcutter bee, *Megachile rotundata* (Rinehart et al. 2016). These leafcutter bees are a great option for use as commercial pollinators because they can be stored in a dormant state, termed diapause—essentially insect hibernation, for up to several months so that bee producers can easily ship these bees to growers, who can subsequently deploy the bees in their fields when needed (Rinehart et al. 2016). This ability for alfalfa leafcutter bees to be stored and shipped in dormancy has been critical for their adoption in agriculture. One limitation is that alfalfa leafcutter bees can only be stored for a limited period of time before they begin to lose efficacy or die, weeks to a few months (Yocum et al. 2021). Yet, some insect species can remain in dormancy for multiple years, such as the solitary desert bee *Perdita portalis*, which frequently remains dormant for 2 years (Danforth 1999), or the apple maggot fly wherein some individuals will enter dormancy of up to 7 years (Boller and Prokopy 1976). Can we leverage knowledge about how these other insect species that can remain dormant for longer do so to engineer longer dormancy in species like alfalfa leafcutter bees? Extending the shelf life of the alfalfa leafcutter bee by even a month or two would have substantial benefits for growers because it would afford them more flexibility in deploying supplemental pollinators as an ecosystem service to maximize their crop yields. But, what new possibilities for the bioeconomy would open up if alfalfa leaf-cutting bees could be stored for 2 years or more like some other insects? Some animal species, like *Daphnia* crustaceans, can survive for decades to centuries in dormancy (Cuenca Cambronero and Orsini 2018; Wersebe and Weider 2023). What new opportunities would open up if alfalfa leafcutter bees could be stored in dormancy for decades or centuries? Sending pollinators to Mars or other planets so agricultural production can occur and support human colonies far away from Earth comes to mind. These examples highlight the promise of engineering radical phenotypes for improving the pollination bioeconomy and for opening up fundamentally new opportunities to study the limits of animal physiology.

For some traits, it may be possible to engineer extreme phenotypes with manipulation of a single endogenous gene or insertion of a single transgene from another species. But, for most complex traits, like bee dormancy, we will need the ability to engineer whole networks in cell-specific manners. Of course, there will be many caveats and constraints to making this work. Pleiotropy and epistasis are among the first concepts taught in many introductory genetics course lectures for a reason: they are ubiquitous effects and will provide challenges to engineering complex genetic constructs that work well in novel genetic backgrounds. Recent work by Storz (2018) and collaborators nicely illustrates how epistatic effects can provide a challenge to novel allelic combinations. Using *in vitro* protein engineering, they showed that there are only a few permissive allelic combinations that can evolve together during adaptation in hemoglobin in the red blood cells of mice to high altitudes due to epistatic effects between alleles across loci within the protein (Cziczo et al. 2013). Similarly, epistatic effects have been shown to alter the penetrance of transgenically engineered traits in model organisms, like *D. melanogaster* flies, wherein the same transgenic construct has very different magnitudes of phenotypic effect across genetic backgrounds (Evangelou et al. 2019). Given this observation, one might expect that approaches involving multiple transgenes, like those we believe will be needed to engineer complex phenotypes across species, will have an even greater chance of epistatic effects and that these effects may not be easily predictable when combining genomic elements that are long separated in evolutionary history. Tackling these challenges will require a systems-level approach to the design and evaluation of novel engineered phenotypes, one that we think is possible in the near-term future given the explosion of computational systems biology driven by both new hardware and software and the use of artificial intelligence (AI) for understanding complex data sets—as we expand upon in the following three vignettes.

Readers should note that this paper about engineering extreme phenotypes came about from discussions between the authors of this paper, as well as many other participants, at the National Science Foundation (NSF)-sponsored workshop LIFE: Leveraging Innovation from Evolution held in Indianapolis, IN during August 2023. We believe strongly that a comparative evolutionary approach can inform the development of new applications that will have benefits for society, but we also believe that the largest societal benefits will ultimately come from support for broad-ranging basic research in phenotypic engineering, including support for high-risk endeavors. While the authors acknowledge that there are many opportunities for modern approaches to phenotypic engineering to improve our understanding of both basic biology and managed systems, we regret that we cannot cover them all. Here, our goal was to selectively provide a few vignettes to stimulate further interdisciplinary discussion across research communities. The first vignette highlights historical examples demonstrating how unexpected basic research has played critical roles in the development and successful deployment of molecular tools that enabled the “first wave” of synthetic biology, reinforcing the need for greater investment in basic science. The second vignette expands on sub-cellular mechanistic perspectives to explore a vision for designer cells, combining knowledge of unusual cell types across the animal tree of life to create novel trait combinations in cells or even whole organisms, illustrating why phenotypic engineering need not be constrained by naturally occurring variations. The third vignette examines how comparative evolutionary approaches can facilitate the expansion of synthetic biology and how these synthetic systems can also inform the basic understanding of evolution, cell biology, and more. The final vignette outlines critical needs for democratizing synthetic biology and engineering of extreme phenotypes to all biologists.

## Naturally selected tools for synthetic biology

(*by Joshua R. Widhalm*)

Synthetic biology is an interdisciplinary field that applies “design, build, test, and learn” (DBTL) principles of engineering to create or modify biological systems for practical applications. The “first wave” of synthetic biology showed us how to combine basic genetic elements (Elowitz and Leibler 2000; Gardner et al. 2000) to design strategies for applications like regulating gene expression (Isaacs et al. 2004), engineering metabolism (Martin et al. 2003), and establishing cell–cell communication (Weiss and Knight Jr 2001). The “second wave” is expected to bring the integration of simple modules to achieve systems-level bioengineering (Purnick and Weiss 2009; Meng and Ellis 2020). Emerging technologies, including but not limited to, expanded computational power, high-throughput phenotyping (Banerjee et al. 2020; Abdollahi et al. 2024), and the use of AI-driven approaches to enhance systems-level understanding (Puniya et al. 2024) are already allowing for better characterization of both natural phenotypic variation and engineered phenotypes. Biofoundries, which are automated modular platforms that integrate synthetic biology and AI, are taking the DBTL cycle to an industrial scale (Dixon et al. 2020). However, there are challenges ahead that will require the implementation of new design principles, improved understanding of the biological mechanisms underlying complex traits, and making sense of unknown “parts” (i.e., genes, enzymes, and regulatory elements) from large-scale systems biology studies (Joshi and Hanson 2024). Thus, as our capacity to engineer accelerates, we will eventually find ourselves limited by a lack of basic knowledge.

As discussed in the section “Introduction,” scientific breakthroughs often emerge not from directly pursuing applications but from curiosity-driven investigations of nature. Here, using historical examples of Nobel Prize-winning fundamental discoveries that led to some of the molecular tools and principles that helped pave the way for molecular biology and the first wave of synthetic biology, I highlight the lasting importance of basic science and the value of finding inspiration from nature.

### The lac operon and M13 plasmid systems

The term “synthetic biology” first appeared in the literature in 1980 (Hobom 1980; Hirschi et al. 2022). However, the principles and tools that shaped the field are rooted in fundamental research published much earlier. Take, for example, the lactose operon (lac operon). In the 1940s, then-graduate student Jacques Monod, who completed his Ph.D. at Sorbonne (the University of Paris) under extraordinary circumstances during World War II (Ullmann 2011), first observed the ability of bacteria to switch sequentially from a preferred sugar source to an alternative once supplies are exhausted (Monod 1942). Following his defense, the laboratory director for whom Dr. Monod worked said, “Monod’s work is of no interest to the Sorbonne” (Ullmann 2011). Undeterred, Monod returned to Paris after the war, this time as a group leader at the Pasteur Institute. Over the next several years, Monod continued to explore the biochemistry and genetics of lactose metabolism in *Escherichia coli*. This work led to his groundbreaking paper with Dr. François Jacob outlining how a simple control circuit called an operon could coordinately regulate a collection of structural genes (Jacob and Monod 1961; Lewis 2011). The concepts first raised by Jacob and Monod were paradigm-changing; they provided the foundational understanding of gene regulation and, unbeknownst to them, would open new fields of biology, including the first wave of synthetic biology decades later. Jacob and Monod, with collaborator Dr. André Lwoff, went on to receive the Nobel Prize in Physiology or Medicine in 1965 “for their discoveries concerning genetic control of enzyme and virus synthesis” (Nobelstiftelsen 2025a). To this day, the lac operon serves as the classic model for teaching the concept of gene regulation in microbes.

Dr. Agnes Ullmann, whom Monod smuggled into France in 1960 to escape persecution in Hungary (Ullmann 2012), was also a key player in researching the lac operon. She is credited with discovering that the lac operon is positively activated by a cyclic AMP-dependent catabolite gene regulator protein (Ullmann and Monod 1968; Lewis 2011). More famously, she discovered the α-complementation model of the β-galactosidase gene (Ullmann et al. 1967), which later became the basis for the blue–white screening method used to identify the insertion of foreign DNA into the lacZα region on plasmids. First introduced into the M13 plasmid system 40 years ago (Vieira and Messing 1982), lacZα-based blue–white screening remains popular worldwide.

Several basic discoveries in phage biology are responsible for the M13 systems at the center of many biotechnologies. The M13 bacteriophage was first identified in the 1960s as part of a broader effort to characterize filamentous phages (Hofschneider 1963). Unlike lytic phages, M13 does not kill its host. Instead, it extrudes through membranes to release new virions while keeping the host alive (Hofschneider and Preuss 1963) and replicates via a double-stranded DNA intermediate (Olsen et al. 1972). This led to manipulating the M13 genome like a plasmid, with key modifications, including a multiple cloning site (Hines and Ray 1980) and the lacZα region (Vieira and Messing 1982), to create the first M13 cloning vectors. Beyond molecular cloning, the M13 phage system helped advance technologies from early DNA sequencing (Sanger et al. 1980; Hindley and Phear 1981) to modern applications in nanotechnology, e.g., (Thibault-Starzyk et al. 2009; Nam et al. 2010; Dang et al. 2011) and CRISPR-Cas9 delivery (Lam et al. 2021). Notably, M13 led to the development of phage display (Smith 1985), which enables the selection and evolution of peptides and proteins by displaying them on the surface of bacteriophages. For this work, Dr. George Smith shared the 2018 Nobel Prize in Chemistry (Nobelstiftelsen 2025b). Phage display has gone on to serve as a building block for applications in biosensing, bioimaging, therapeutics, and energy harvesting (Wang et al. 2023).

### Type-II restriction enzymes

As a new assistant professor of microbiology at Johns Hopkins University, Dr. Hamilton Smith set out to continue his work on bacteriophages. Before joining the faculty, Smith spent several years at the University of Michigan studying genes controlling lysogenization and prophage attachment (Kresge et al. 2010). What he and his new graduate student, Kent Wilcox, instead found through their investigations on bacteriophage recombination in *Haemophilus influenzae* was an enzyme called “R” (Kelly and Smith 1970; Smith and Wilcox 1970). Today, we know “R” as the Type II restriction enzyme *Hin*dII. It was by chance that a week before Wilcox joined the lab and began doing DNA uptake experiments to study recombination, Smith presented a paper at a journal club on a recently discovered Type I restriction enzyme. When Wilcox could not recover the foreign DNA from the cell following uptake, Smith made the connection that perhaps it could be due to restriction (Gitschier 2012). It was a radical idea at the time, as restriction had only been described in *E. coli*. It was unknown that restriction enzymes broadly serve as defense mechanisms against viruses in bacteria and archaea. Indeed, subsequent experiments independently verified the presence of endonuclease activity (Gitschier 2012), and Smith, alongside Drs. Werner Arber and Daniel Nathans, later received the 1978 Nobel Prize in Physiology or Medicine “for the discovery of restriction enzymes and their application to problems of molecular genetics” (Nobelstiftelsen 2025c). Smith and others recognized early on that restriction enzymes could, beyond their biological roles, serve as powerful research tools, and as such, they were instrumental in developing recombinant DNA technology (Pederson 2017).

### CRISPRs

Dr. Yoshizumi Ishino is credited with discovering the first clustered regularly interspaced short palindromic repeats (CRISPRs) over 35 years ago. As a new postdoctoral researcher at Osaka University in Japan, he was studying the gene responsible for isozyme conversion of alkaline phosphatase in *E. coli* (Ishino et al. 1987). His work would lay the foundation for CRISPR-guided tools a quarter of a century later (Wiedenheft et al. 2012). It was after CRISPRs were found to be conserved across bacteria and archaea (Mojica et al. 1993) and the discoveries of CRISPR-associated (*cas*) genes (Jansen et al. 2002; Makarova et al. 2002) and sequence similarities between CRISPR spacer regions in bacteriophages, archaeal viruses, and plasmids (Bolotin et al. 2005; Mojica et al. 2005; Pourcel et al. 2005), that the importance of CRISPR-Cas as a prokaryotic immune system (Barrangou et al. 2007; Gasiunas et al. 2012; Jinek et al. 2012) and its translational potential for genome editing (Cong et al. 2013; Mali et al. 2013) were recognized. Drs. Emmanuelle Charpentier and Jennifer Doudna, two of the pioneers in unlocking the biological mechanisms underlying CRISPR technology, would go on to share the 2020 Nobel Prize in Chemistry (Nobelstiftelsen 2025d) for their discoveries. Since the development of CRISPR-Cas9-mediated genome editing just over a decade ago, applications of the technology have transformed biological research, medicine, and agriculture (Wang and Doudna 2023).

### Looking to the future

Scientific discoveries rarely follow a linear path, and any transformative impacts on society often become clear only in hindsight. Breakthroughs in biology have often resulted from a curiosity to understand how life works. When Dr. Ishino identified CRISPRs back in 1987, it was impossible for him to predict the biological function of the cryptic sequences; he said so himself (Ishino et al. 2018). Yet, decades later, his discovery launched a revolution in genome editing. Likewise, early work on bacterial gene regulation by Drs. Monod, Jacob, Ullmann, and others laid the foundation for key concepts in synthetic biology long before the field even had a name.

As we enter the next phase of synthetic biology and try to understand how life emerges from its integrated parts, we should remember that the innovations of tomorrow will likely depend on today’s investment in basic science. While it is tempting to shift focus and funding toward application-driven research, we must continue to draw inspiration from nature. Otherwise, we risk constraining our options to only what we can conceive or by what a machine can reason. That does not mean, for example, we should not embrace the roles of machine learning for predictive metabolic engineering (Lawson et al. 2021) or biofoundries to automate biological design and testing (e.g., Vickers and Freemont 2022). Such approaches in combination with a commitment to a curiosity-driven understanding of nature will accelerate the rates of discovery and innovation to ensure the future of synthetic biology is both transformative and firmly rooted in the fundamental principles of life itself.

## The weird and wonderful world of novel cell types

(*by Leslie S. Babonis*)

Evolution has produced cells that defy the imagination. Cells in the spinning glands of spiders produce silk fibers that are stronger than steel (Debabov and Bogush 2020). Cells lining the cavity of a deep-sea sponge make glass fibers with optical properties that rival synthetic fiber optics (Fernandes et al. 2021). Cells in the hinge of a mussel shell secrete collagen-based threads that have the tensile strength of Kevlar (Areyano et al. 2022). The list goes on. More than just curiosities of biology, these traits have emerged as an important source of inspiration for many practical applications and fueled numerous advancements in the bioeconomy. For example, hagfish mucous cells produce copious slime with gel-like properties that are revolutionizing the food industry (Böni et al. 2016), and the harpoon-laden stinging cells of sea anemones have been co-opted as a tool for transdermal drug delivery (Lotan 2016). The cells that express these bizarre phenotypes have arisen naturally as evolutionary novelties—taxon-specific traits that perform a unique function (Moczek 2023). Now, with access to genetic engineering technology, we have the power to make our own novel cell types by bringing together genes that have never come together naturally. We simply need to decide which traits to combine.

### Mix-n-match cell types

Eukaryotic cells have been diversifying for 2 billion years and it is hard to imagine what cell functions have not yet evolved. There are cells that can survive desiccation (Boothby et al. 2017), and there are others that even permit other cells to live inside them (Muñoz-Gómez et al. 2021). Not all cells can do these things. In fact, there are many cells that can do none of these things, but why? Because they lack a fundamental property or because they have never been given the chance to express these phenotypes? At the individual or species level, phenotypic evolution is limited by historical contingency (e.g., whales will never have gills because they evolved to be aquatic from a terrestrial, lung-bearing ancestor), and cell phenotype is limited in the same way. The cells of a spider, for example, will never have the chance to express the genes that arose uniquely in a jellyfish. Or will they? The development of a wide array of techniques for editing genomes means that we are no longer limited by the phenotypes that evolution has produced for us. We are free to mix and match genes across species boundaries and generate fundamentally new phenotypes.

Evolution has been playing the mix-n-match game for a long time. Horizontal gene transfer (HGT)—the transfer of genes from one organism to another—occurs naturally in many systems and serves as an important source of novel genes in a genome. Although these events frequently result in the transfer of genes between viruses and eukaryotes (Irwin et al. 2021) or bacteria and eukaryotes (Husnik and McCutcheon 2018), transfer among eukaryotes is also known to occur (Van Etten and Bhattacharya 2020). Like many other aspects of evolution, however, the gain of a novel trait through HGT is thought to be uncommon and, thus, unpredictable. With the advent of genome editing technology, we are no longer constrained by the slow pace of evolution; introducing a gene from a sponge into a silkworm or a gene from a hagfish into a coral is relatively straightforward and can enable traits to come together that would otherwise never naturally meld. The challenge is that experiments like these are risky—what if they do not work? What if they work as expected and produce a phenotype that cannot be characterized or that we do not yet know how to use? What good is a coral that makes hagfish slime? What do we do with a caterpillar that spins glass fibers? These are good questions and although they may lack answers (for now), we should not allow ourselves to be held back by the limits of our imaginations. We have tools that can fundamentally change the starting point of evolution, and we should consider it our duty to see where these tools can take us. Science is already exploring various avenues for manipulating the functions of cells through the development of designer, membrane-free organelles and by co-option of a secretory cell to secrete a novel product (see below). How far can we take this?

### Designer, membraneless organelles

Efforts to develop designer, membrane-free organelles have expanded in recent years since the discovery of intracellular phase separation (Antifeeva et al. 2022). Aggregates of intrinsically disordered proteins create subcellular regions that can sequester other proteins through interactions with disordered protein domains. Adding a disordered domain to a native protein using CRISPR will target the molecule to such an “organelle,” effectively sequestering it away from where it normally functions and permitting the control of basic cell functions like the timing of division (Garabedian et al. 2021). The development of optogenetic techniques that promote aggregation of intrinsically disordered domains using a light cue enables fine, temporal, and spatial control of protein localization (Shin et al. 2017). Using this technique, it becomes possible to concentrate transcriptional cofactors (Sabari et al. 2018) or regulate the translational machinery (Reinkemeier et al. 2019) to modify gene expression. This ability to manipulate the intracellular environment on a per-cell basis is unprecedented—What do we do with this technology? Taking advantage of the ability to generate a diverse array of membraneless organelles in the same cell (Welles et al. 2024) could give us the power to create subcellular bioreactors that can simultaneously generate multiple components of a multi-part product (like a 2-part epoxy). Maybe we can redirect neurons into novel relationships by modifying the cues that induce synaptic transmission (Guzikowski and Kavalali 2024) or changing the behavior of the synaptic vesicles (Qiu et al. 2024). Membraneless organelles are already playing a role in directing chromatin conformation (Hirose et al. 2023); maybe by modifying these organelles we can direct the physical interactions between chromosomal loci to coordinate the co-expression of novel gene networks. The power contained in controlling the function of membraneless organelles seem boundless, much like the organelles themselves.

### Co-option of the secretion machinery through genome editing

The secretory vesicle is the ultimate renewable resource. For example, just 30 min after release of insulin, pancreatic beta cells have fully regenerated their vesicles (Vasiljević et al. 2020). All eukaryotic cells have the capacity to secrete products into the extracellular space. The origin of regulated secretion—the ability to package and store products in intracellular compartments for cued release—was a significant innovation in the evolution of eukaryotes. The gain of the cued secretory vesicle ultimately permitted the evolution of a wide variety of specialized animal cell types, including mucous cells, exocrine cells, and neurons. Figuring out how to clear a secretory cell of its native cargo and replace it with an alternative cargo would create practical applications for biomaterial production. This has already been accomplished on a single-gene basis. Using CRISPR, a recent study swapped the native fibroin gene (the primary component of silk) from a spider into a silkworm (Mi et al. 2023). While the silk fibers of spiders are mechanically superior to those of silkworms, the production of these fibers from silkworms far exceeds that of spiders. Using genome engineering, we can potentially have the best of both worlds and open up the possibility of developing new biomaterials. Can we express the stretchy, elastic fibers of a jellyfish’s stinging cell (“cnidoin”) in silkworms? The evolutionary distance between silkworms and jellyfish is far wider than that of silkworms and spiders (jelly fish and arthropods last shared a common ancestor ∼800 million years ago whereas arthropods diversified into arachnids and lepidopterans ∼300 million years later), but the functional domains of fibroin and cnidoin are highly similar. Does the cell care what protein it packages and stores for secretion or is the silkworm’s secretory cell an untapped resource for mass production of whatever protein we want? Could we enlist these cells to make glass fibers, rather than silk fibers, by introducing a sponge gene into the silkworm’s genome? Experiments like these hold the key to developing radical new phenotypes that could change our understanding of cell type evolution and produce fundamentally new and unpredictable innovations.

### Where are we going?

Ongoing efforts to develop artificial cells from component parts offer the ability to make cells that have never evolved naturally—cells that defy the limitations of the physical constraints of our current environment. In theory, we could design artificial cells that could withstand the desiccating climate on an exoplanet or the vacuum of space. In accomplishing these feats, we would learn fundamentally new things about cell biology and about the limits of cell type evolution. The challenges of creating synthetic cells that replicate functions that have evolved naturally among living cells are non-trivial, and in many ways, it may be more feasible to let natural cells do their work in a new context. Much of what we might like these synthetic cells to do for us has already evolved among taxa that might have gone overlooked. Want cells that proliferate rapidly but controllably to facilitate regeneration? Flatworms can help with this (Vila-Farré et al. 2023). Reid and Latty (2016) want a cell that will harpoon and kill another cell to take down cancer before it becomes a problem? Dinoflagellates have this all worked out (Gavelis et al. 2025). What about a cell that will remove microplastics from the ocean? Yep—there are bacterial cells that will do that for you (Li et al. 2023). With enough time and tinkering, we can probably design artificial cells that could do each of these jobs. Or, we could just start with cells that evolution has made for us and mix their instruction manuals.

## Artificial or synthetic cells as an experimental paradigm for studying evolution

(*by Allen P. Liu*)

A fundamental question in biology is how genotypes as inherited materials relate to phenotypes as observable attributes. Addressing this profound question can help us understand how phenotypic diversity arises, getting to the heart of the question of what makes species different from one another or even what generates individual-level variation within populations. Building an understanding of the genotype–phenotype map also helps illuminate the core principles of evolution—variation, heredity, and differential fitness (Papale et al. 2020). Comparative genomics within and across species has enabled researchers to systematically explore evolutionary processes that lead to phenotypic differences between species, as well as to understand the structure and function of genes (Bornstein et al. 2023). Yet, there remain several gaps in knowledge with regard to the evolution of single-celled organisms. One of those outstanding questions is, what are the minimal components needed for a cell to function? In this vignette, I first briefly discuss a minimal cell approach, followed by introducing the concept of using artificial or synthetic cells constructed from the bottom up as an experimental platform for systems-level evolution. I will close the section with a brief discussion on the potential for AI-driven approaches to artificial cell development and metabolic engineering of living cells for novel phenotypes.

The ultimate goal of taking a reductionist approach to dissect biological systems into constituent parts is to determine the connection between the parts to understand the whole system. Venter and coworkers, in 2016, constructed a bacterial cell that encodes *only* the genes necessary for cellular life (Hutchison et al. 2016). Whole-genome design and synthesis from chemically synthesized oligonucleotides were used to minimize the 1079-kilobase pair synthetic genome of *Mycoplasma mycoides*, with the original synthetic genome termed JCVI-syn1.0 (Gibson et al. 2010) by their team and the reduced genome strain known as JCVI-syn3.0 or syn3.0. The new genome-reduced organism syn3.0 has 473 genes, smaller than any autonomously replicating cell found in nature (Hutchison et al. 2016). Such an astonishing feat of creating a minimal cell in biology is akin to studying the simple hydrogen atom in physics/chemistry, and this new minimal cell is key to paving the way to understanding the first principles of cellular life (Glass et al. 2017). Genes in the minimal genome can be partitioned into four major functional groups: expression of genome information, cell membrane structure and function, cytosolic metabolism, and preservation of genome information, in the order of their abundance (we will come back to this later when discussing artificial cells). What was surprising is that 79 genes (or 17%) have no assigned functional category. For obvious reasons, these genes of unknown function that are required in syn3.0 *must* represent nearly universal functions in cellular biology. While genome reduction to achieve a minimal cell is a triumphant success, this is only the starting point. We imagine two critical next steps: First, to decipher what these genes of unknown functions to support life are; second, and more importantly for the context of this vignette, to use the minimal cell as a basis to evolve toward greater complexity, allowing the field to test evolutionary theories linking genotype to phenotype using this minimal cell model system (Moger-Reischer et al. 2023).

Interestingly, the minimal cell syn3.0 exhibited greater morphological variation, including filamentous, vesicular, and irregular shapes, compared to the original synthetic *M. mycoides* strain, JCVI-syn1.0 (Gibson et al. 2010). By introducing 19 genes across eight gene clusters, Glass, Strychalski, and coworkers generated JCVI-syn3A or syn3A that restores normal morphology in JCVI-syn1.0 (Pelletier et al. 2021). They achieved this by using a reverse genetics approach wherein the investigators inserted genes into their synthetic minimal genome and performed microfluidic imaging of cellular growth and morphology to identify the minimal synthetic genome to restore a nearly wild-type free-living mycoplasma morphology. Of the 19 additional genes added in syn3A, seven were required together to restore a morphological phenotype similar to that of wild-type mycoplasma. Two of the seven are known cell division genes, one is a hydrolase, and four genes encode membrane-associated proteins of unknown function—reinforcing the challenge in linking genes to function, even in a minimal organism. This study highlights the use of genomically minimal cells to study key parameters of cell physiology, including cell shape and division.

A fundamental feature of cellular life is the membrane. With 18% of the syn3.0 genome encoding proteins related to cell membrane structure and function, it is clear that membrane synthesis and assembly followed by molecular traffic across the lipid membrane via transporters and/or pumps are crucial processes in a minimal cell. This begs the question: what is the minimal lipidome that can sustain life? Interestingly, mycoplasmas have evolved to acquire membrane components directly from their environment (Breuer et al. 2019), thus making syn3A ideally suited for exploring the minimal cellular synthetic requirements for membrane biology. In an elegant study, Justice and Saenz, using syn3A, demonstrated that two lipids, diether phosphocholine and cholesterol, are sufficient to sustain the minimal cell, although these cells had a far-from-optimal membrane lipidome because their growth rates were much lower than cells provided a more complete set of lipids for use in membrane assembly (Justice et al. 2024). This fascinating system allows for a tunable minimal membrane system, where complexity can be systematically introduced to explore the role of lipid composition in supporting cellular functions—shedding light on why life has evolved to utilize so many lipids.

While studying minimal cells with only essential genes will surely make an impact on revealing mechanisms and processes required for autonomous cellular life, it is not unreasonable to postulate that the first living cell (however one wishes to define a cell) contained even fewer than 473 genes. A complementary approach to using minimal cells is to construct compartmentalized biochemical systems that resemble key features of a cell. If the right ingredients are included, it is perhaps possible that the four major functional groups of proteins found in a minimal cell (for expression of genome information, cell membrane structure and function, cytosolic metabolism, and preservation of genome information) could be constituted with even fewer components than the minimal cell. This is a grand and formidable challenge in building artificial or synthetic cells from the bottom up (Noireaux and Liu 2020; Stano 2022; Adamala et al. 2024) and may be more appropriate for addressing the origin of cellular life rather than the approach of making minimal functional cells by deleting components in existing systems.

On one spectrum of synthetic cell systems are protocells that consist of a fatty acid membrane that compartmentalizes an informational polymer capable of replication and inheritance (Schrum et al. 2010). Protocells are often described as primitive cells with the potential for classical Darwinian biological evolution to help explore potential pathways for the emergence of life on the early Earth. A core assumption of the RNA world hypothesis for the origin of life is that an RNA genome of a primitive cell can be replicated by a ribozyme RNA polymerase (Gilbert 1986). While nonenzymatic RNA copying is possible, the high magnesium ion concentration required for RNA copying is incompatible with fatty acid membrane stability. To address this, Adamala and Szostak (2013) found a solution to this longstanding problem by discovering that the magnesium chelator citrate effectively protected fatty acid vesicles while allowing magnesium to drive the assembly of the RNA molecule. Prebiotic compartmentalization studies that involve liquid–liquid microphase separation via the formation of coacervate microdroplets have been central to providing insights into how protocellular systems may have emerged on the early Earth, but such systems lacked lipid membranes. Taking simple chemical and physical processes further in driving the emergence of protocellular systems, as might have been expected on the early Earth, Tang, Mann, and coworkers demonstrated spontaneous self-assembly of a continuous fatty-acid membrane at the surface of preformed coacervate microdroplets (Dora Tang et al. 2014). This hybrid protocell model has a membrane that mediates selective uptake or exclusion of small and large molecules and supports the foundation for building coacervate organelles in protocell models (Deng and Huck 2017; Choi et al. 2022). While an undeniably useful tool, the simplistic nature of RNA-based protocells puts a limit on the ability of these systems to relate genotypes to phenotypes with regard to membrane stability and RNA replication. As we know of the life of “modern” cells, DNA is used as a hereditary molecule, with RNA serving as an intermediate molecule. Thus, artificial cell systems that employ DNA, machinery to process DNA, and produce proteins are much more powerful experimental platforms for investigating the genotype–phenotype relationship and testing evolution theories, whereas RNA-based protocells are well suited to exploring the origins of cellular life.

Built on the foundation of the central dogma of molecular biology, the link between genotype and phenotype can be interrogated in an artificial cell by either using transcriptionally and translationally active cell-free lysates (Noireaux and Libchaber 2004) or with purified translation machinery commonly known as PURE (protein synthesis using recombinant elements; Shimizu et al. 2001). Earlier work has utilized such a platform for understanding the molecular evolution of a protein or RNA with a desired catalytic activity by linking a substrate to a gene that encodes the protein or RNA in compartments formed from water-in-oil emulsions (Tawfik and Griffiths 1998). However, expression of multiple genes in a controlled manner with predictable dynamics is necessary for establishing a more complex genotype–phenotype relationship. By employing dual-gene expression assays with two fluorescent reporter proteins inside lipid vesicles, it was shown that there was no linear correlation between the amount of encapsulated DNA templates and the level of fluorescence reporter proteins (Nourian and Danelon 2013). Although this may seem counterintuitive at first, this finding is attributed to the stochastic encapsulation of enzymes in vesicles—one can imagine a vesicle containing many DNA molecules but lacking RNA polymerase would not yield gene expression. The stochastic encapsulation of biomolecules in lipid vesicles gave rise to significant heterogeneity in fluorescence outputs of the two reporters and, thus, heterogeneity in gene expression. With this caveat, how can an artificial cell platform be amenable to investigating genotype–phenotype relationships given the vesicle-to-vesicle heterogeneity? We need a new approach that embraces heterogeneity.

We believe a systems-level evolution approach is a powerful alternative to rational engineering. This concept was nicely described by Abil and Danelon (2020). Information (i.e., DNA and RNA) and molecular hardware (i.e., proteins, lipids, cofactors, etc.) by themselves do not define life but rather functions that emerge from their interactions and, importantly, with the environment would yield attributes that define life (i.e., life is a system of interactions). Complex phenotypes typically do not emerge from variants in single genes, even in single-celled organisms. Instead, life includes interactions of gene products in multifaceted biochemical or cellular pathways to generate phenotypes and are subject to the landscape of pleiotropy and epistasis (Xu et al. 2012). Thus, we must consider the interactions among components in these networks to understand the genotype–phenotype map in synthetic cellular systems. A series of genetically encoded processes, including anabolism, catabolism, and various feedback and regulatory mechanisms, are expected to co-evolve with each other in an artificial cell to confer some phenotypic advantage in a particular environmental condition. Thus, the premise of a systems-level approach involves iterations of functional module integration, genetic diversification, and phenotype assessment. Successful integration of functionalities that can be tuned by selection is expected to gradually increase the complexity and autonomy of an artificial cell toward life. By engineering systems that have the potential to accumulate heritable variation that is subject to selection, we can facilitate an evolutionary process in synthetic cellular systems. This ability of synthetic systems to accumulate heritable variation that can lead to differential survival reproduction and, thus, undergo selection, will provide the opportunity for us to test the extent to which convergent evolution may occur between synthetic and natural systems.

Adaptive evolution in an artificial cell is an important step toward mimicking living systems. As a proof-of-concept study, Abil et al. (2024) recently devised a scheme for a synthetic DNA self-replicator capable of undergoing Darwinian evolution to accumulate mutations conferring a selection advantage over ten rounds of evolution. Sustainable replication of a linear DNA template that encodes for DNA polymerase and terminal protein from the Phi29 bacteriophage that together form the self-replication machinery was achieved. Importantly, they also demonstrated adaptive evolution via cycles of compartmentalized *in vitro* transcription–translation–replication using the PURE system. Mutations that were accumulated increased replication ability within liposomes, a systems-level function that necessarily includes interactions among inherited components (i.e., genes and gene products). This is a major advancement in a systems-level evolution approach for artificial cells that lays the foundation for co-evolving more complex cellular functions *in vitro* through Darwinian evolution.

Systems-level synthetic cell phenotypes (without a genotype) can also be illustrated with synthetic cells built from purified proteins in a simple biochemical system, as opposed to a system with heritable genetic information. Bashirzadeh et al. (2021) reconstituted actin cytoskeleton networks in giant unilamellar vesicles by encapsulating actin and actin-binding proteins revealed unexpected systems-level complexity. Even in the simplest system of actin with two different types of crosslinkers, fascin and alpha-actinin, there is a rich phenotypic space of actin network architectures that depends on both the confinement size as well as the relative proportion of the two crosslinkers (Bashirzadeh et al. 2021). How can we bridge the gap between the simplified biochemical systems and the complicated whole organisms in establishing a genotype-to-phenotype relationship? How can we realize saltatory jumps in phenotypic space by extending the conceptual framework of systems-level evolution? The use of synthetic cells can play a key role here. Instead of starting from protocells with primitive life-like properties, evolving synthetic cells with a synthetic genome and some amount of internal complexity (i.e., organelles or cytoskeleton networks) can be a starting point for evolving systems-level functionalities. Ultimately, to facilitate large changes in cell function via synthetic biology approaches, we will need to integrate many genes acting in networks, including autoregulatory and feedback networks. Providing the ability for the components of those networks to accumulate heritable variation that can be acted on by selection can connect the generation of novel phenotypes via synthetic biology approaches to naturally evolved phenotypes and test the extent to which synthetic systems accumulate convergence with existing natural systems across levels from genes to networks to cellular phenotypes, and the extent to which evolution solves problems differently in synthetic systems than we have observed in natural systems.

Although I have restricted my discussion to systems-level evolution of artificial cells, the same systems-level framework applies to the evolution of organisms that have radical phenotypes. The complexity of biosystems with interconnected metabolic pathways and enzymes makes it an extraordinary challenge for systems-level engineering of novel phenotypes in organisms. In recent years, machine learning (ML) and deep learning (DL) have helped scientists make substantial progress in synthetic biology (Goshisht 2024). ML/DL models have successfully been applied to cell and metabolic engineering (Tunney et al. 2018). Integration of data-driven methods with genome-scale metabolic models allows for accurate prediction of microbial bioproduction from engineered *E. coli* (Oyetunde et al. 2019). Combined with high-throughput technologies, including robotic liquid handlers and high-throughput, high-content imaging that are available in biofoundries and are increasingly more common in research labs, the engineering of cell factories aimed at generating a wide range of extreme phenotypes is within reach. In the context of artificial cells, the combination of high-throughput droplet microfluidics and AI-based screening can aid the discovery of conditions beyond the experimental scale to predict high-yield formulation of a cell-free expression systems (Zhu et al. 2025). Technological advances in automation and ML/DL will continue to fuel the second wave of synthetic biology and shape the future of contributions of synthetic biology to the bioeconomy.

## The road ahead: investments needed to engineer radical phenotypes

(*by Daniel A. Hahn and Joshua R. Widhalm*)

Here, we have laid out a grand vision of being able to engineer radical phenotypes that are not currently found in nature. These novel radical phenotypes can be used to both stimulate the bioeconomy through new product development and to answer fundamental questions about organismal function and limits to trait evolution in nature. What would it take to make our grand vision of using synthetic biology techniques to engineer extreme phenotypes and emergent traits a reality?

### Increased funding for basic science

As we enter the “second wave” of synthetic biology, we must reflect on what made the field successful in the past (Purnick and Weiss 2009; Meng and Ellis 2020) and draw lessons for the future. There are many challenges ahead. Systems biology continues to supply potential “parts” to fuel synthetic biology efforts. However, the functions of most parts remain unknown (Reynolds et al. 2021), and in most cases, there is no metaphorical instruction manual for how the parts fit together or can be manipulated (Joshi and Hanson 2024). Understanding the function of a particular gene is only the first step. Going forward, researchers must also consider how a part will behave in the context of a living organism, how its expression affects the expression of other parts and cellular processes, and how the environment influences those interactions. Synthetic biologists must also account for coordinated cellular behavior, cell death, and dynamic intracellular, intercellular, and extracellular conditions in multicellular systems. We need to study the mechanisms that organisms use to recognize and interact with one another and respond to changing environments (Purnick and Weiss 2009). We then need to achieve desired phenotypes in larger and more complex systems without placing heavy physiological burdens on the engineered organisms (Cameron et al. 2014). Ultimately, we will require fundamental understanding of how life itself emerges from the collection of its parts.

The translational applications that arose from the discoveries of the *lac* operon, M13 phages, restriction enzymes, and CRISPRs were not the result of targeted initiatives for short-term practical gain but the outcome of curiosity-driven explorations by scientists devoted to their fields. These were not sure bets sought after by large businesses. Small, collaborative groups made the seminal discoveries. In many cases, the pioneers were graduate students and early-career researchers pursuing fundamental knowledge for the sake of understanding. This pattern is no coincidence. Small teams are the disruptors in science and technology (Wu et al. 2019). This raises the question of whether investing in a handful of larger bold, audacious endeavors or a greater number of small groups is wiser. The answer is probably to support a range of team sizes (Wu et al. 2019), but we should not sacrifice one for the other. We need to invest *more* across the board to fuel the basic science that will lay the foundation for the bioeconomy.

### Thinking long term

The freedom to explore uncharted territories in basic science has consistently yielded disproportionate returns, leading to innovations that have transformed entire fields (Flexner and Dijkgraaf 2017). The most profound impacts, however, often emerge decades later as we gather the knowledge needed to transform once-obscure discoveries into groundbreaking innovations that can lead to products. Take the timeline of CRISPR-Cas technology, for example. The first applications of CRISPR-mediated genome editing emerged more than 25 years after the initial discovery of CRISPRs and only after multiple subsequent curiosity-driven investigations into prokaryotic immunity made by many groups. Are we positioned to fund basic research today to enable the technologies of tomorrow? Unfortunately, it seems we are eating the proverbial seed corn.

Without adequate support for basic science, research and development (R&D) in the private sector will eventually become stagnant. Recognizing the long arc from discovery to innovation, the US Federal Government has historically played a critical role in supporting basic science, though support from key agencies like the NSF is shrinking. Up until recently, grants from the NSF accounted for about 25% of the federal support to America’s colleges and universities for basic research, but in 2024, lawmakers imposed an 8.3% cut, reducing NSF’s budget to USD $9.06B (Brainard et al. 2024). At the time of writing this piece, even more drastic cuts are expected in 2025. Neglecting investment in basic science risks missing the discoveries that will lead to the next high-return breakthroughs. The global market for CRISPR-Cas technologies, for example, was estimated to be USD $4.7B in 2024 and is projected to reach USD $11.3B in 2030 (Grand View Research 2024). Strengthening public funding for fundamental research is, therefore, not only a scientific imperative, but also an economic one.

In 1964, the last peak in the amount of money that went into US R&D relative to gross domestic product (GDP), total (federal + business) R&D expenditures accounted for 2.79% of GDP. Of that, two-thirds, or 1.86%, came from the federal government. In 2021, total R&D expenditures were up to 3.40% of GDP, but less than one-fifth, or 0.66%, came from the federal government (Anderson and Moris 2023). Thus, US businesses today account for 73% of domestic R&D, and the federal government is investing one-third of what it did in 1964 as a fraction of GDP (Anderson and Moris 2023). On the surface, total expenditures on R&D are up. This is good. However, most R&D development from the business sector is on the applied and development side (Souvaine 2019) and is under pressure to deliver short-term outcomes and rapid financial gains. Thus, the increasing trend toward a free-market approach benefits the later stages of innovation to produce products but is less conducive to the basic science that leads to the initial steps facilitating novel product development.

### Supporting the training of more curiosity-driven students

We are also missing opportunities to train the next generation of basic scientists in the US In 2021, the US Federal Government supported 15% of full-time science and engineering graduate students (primarily doctoral degree students) across all federal agencies, a decline from the most recent high of 21% in 2004 (National Science Board 2024). Of the 42% of basic research funded by the federal government, approximately half is conducted at higher education institutions (Souvaine 2019). Thus, only a small percentage of doctoral students are federally supported to do basic research. Surely, others are participating and supported through other means, like teaching assistantships. However, the point remains that too few of our students are engaged in curiosity-driven science. Federal funding to support doctoral students in basic research needs to increase to ensure the scientific workforce of the future is trained to provide the foundational knowledge needed to drive future innovation.

### Investment in infrastructure

We must invest in shared resources that reduce barriers for investigators to apply genetic engineering and synthetic biology approaches to their systems of interest. One mechanism that has been particularly effective for some communities to adopt genetic engineering and synthetic biology techniques are centralized facilities dedicated to providing training and services to investigative groups that would like to try these techniques in their particular systems and bring the technology successfully to their labs. For example, the University of Maryland’s Transgenic Insect Production Facility has allowed many labs to move into producing transgenic insects for their research needs. This facility provides a combination of genetic engineering design consultation, construct creation and microinjection services, along with providing other services and infrastructure, effectively lowering the barrier for many labs wishing to enter insect genetic engineering. With support from NSF, NIH (National Institutes of Health), and the National Institute of Standards and Technology (NIST), as well as the University of Maryland, this facility’s training courses and services have made genetic engineering available to many programs working on insects. Similarly, the University of Florida Crop Transformation Center was recently founded to translate tools and knowledge of genetic engineering initially developed in model crops like maize and soybean to other plants with the potential for developing new cultivars with favorable traits to allow agricultural production to keep up with demand from the rapidly expanding human population. There are a handful of such facilities offering training and infrastructure that we believe will help us reach our grand vision for phenotypic engineering, and we believe that support for more such facilities would have the effect of democratizing the tools needed to accelerate research innovation and provide novel ideas and products to support the bioeconomy.

### Final remarks

The future of science, and the economic benefits that have time and time again been shown to accrue from basic research, depend on our willingness to invest in the unknown. The examples we give throughout this piece demonstrate that basic science is not just a foundation, but the engine of discovery that ultimately leads to economic and societal benefits. This is not just true for biology, but for all fields of science.

We firmly believe that our grand vision of making engineering of organisms with extreme phenotypes routine and commonplace can be achieved within a decade or so, given adequate support, despite identifying some technical and resource-availability challenges in this paper. What support do we believe is needed to make this grand vision a reality? First and foremost, the field will require increased investment in supporting research teams. Programs like NSF’s Enabling Discovery through Genomics (EDGE), the NIH’s National Institute for General Medical Sciences (NIGMS), and the NIH Director’s Pioneer Awards have been critical for supporting basic science efforts and facilitating discoveries leveraging naturally occurring variation in important organismal traits and have paved the way for our grand vision. NIH’s Pioneer Awards even encourage the kinds of creative approaches that we advocate for here. Similarly, some private foundations have funded the kinds of transformative work consistent with our grand vision. However, to make a large step toward achieving our grand vision, more resources for awards through these programs or new programs will be urgently needed. Because of the potential for the engineering of extreme phenotypes to provide substantial gains to the bioeconomy, we are also hopeful that private–public partnerships could be expanded, such as the partnerships represented by the NSF’s Industry-University Cooperative Research Centers (IUCRC) program. Some Small Business Innovation Research (SBIR) programs supported by a variety of federal agencies could also be instrumental in later stages of developing engineered phenotypes as they move towards commercialized products. Overall, more investment in supporting a range of investigators, from individual labs to small groups to larger consortia, in the initial exploratory stages of R&D is necessary to provide the substrates to generate products for the bioeconomy.

As the field of synthetic biology grows, it is essential that we continue to draw inspiration from nature, exploring and investing resources and effort into understudied organisms that naturally achieve the outcomes we seek to replicate in our phenotypic engineering framework. Doing so can unlock new biological mechanisms and design principles that will propel us forward. To ensure this promise, we must recommit to investing in basic science. We encourage funding agencies to prioritize curiosity-driven research and support more individual principal investigators pursuing novel ideas, even if these ideas are a bit risky. With increased investment in basic research, especially from the federal government, we can empower a new generation of scientists. If the past is prologue, this steady approach to funding basic research will unlock the next wave of transformative innovations to help us tackle the grand challenges ahead.

## Author contributions

All authors contributed to conceptualization, writing the original draft, and reviewing and editing the manuscript.

## Notes

From the National Science Foundation “LIFE: Leveraging Innovations from Evolution” scoping workshop, August 14-16, 2023.
